# Trimethylamine increases intestinal fatty acid absorption: *in vitro* studies in a Caco-2 cell culture system

**DOI:** 10.1017/jns.2023.91

**Published:** 2023-11-03

**Authors:** Catarina Rodrigues, Shámila Ismael, Inês Castela, Inês Barreiros-Mota, Maria João Almeida, Gilberto Maia Santos, Conceição Calhau, Júlio César Rocha, Ana Faria, João R. Araújo

**Affiliations:** 1 Nutrition & Metabolism, CHRC, NOVA Medical School, Faculdade de Ciências Médicas, NMS, FCM, Universidade NOVA de Lisboa, Lisboa, Portugal; 2 Nutrition & Metabolism, CINTESIS@RISE, NOVA Medical School, Faculdade de Ciências Médicas, NMS, FCM, Universidade NOVA de Lisboa, Lisboa, Portugal; 3 Unidade Universitária Lifestyle Medicine José de Mello Saúde by NOVA Medical School, Faculdade de Ciências Médicas, NMS, FCM, Universidade NOVA de Lisboa, Lisboa, Portugal; 4 Reference Centre of Inherited Metabolic Diseases, Centro Hospitalar Universitário de Lisboa Central, Lisboa, Portugal

**Keywords:** Caco-2 cells, Enterocytes, Fatty acid absorption, Trimethylamine, EDTA, ethylenediaminetetraacetic acid, F, forward, FABP, fatty acid-binding protein, FABPpm, plasma membrane fatty acid-binding protein, FAT/CD36, fatty acid translocase, FATP4, fatty acid transport protein 4, FBS, foetal bovine serum, FSA, fluorescein sulphonic acid, HPRT, hypoxanthine guanine phosphoribosyltransferase, MTT, 3-(4,5-dimethyl thiazol-2-yl)-2,5-diphenyl tetrazolium bromide, PBS, phosphate-buffered saline, qRT-PCR, real-time quantitative reverse transcription polymerase chain reaction, R, reverse, sem, standard error of the mean, TG, triacylglycerol, TMA, trimethylamine, TMAO, trimethylamine *N*-oxide

## Abstract

Although elevated blood levels of trimethylamine *N*-oxide (TMAO) have been associated with atherosclerosis development in humans, the role of its gut microbiota-derived precursor, TMA, in this process has not been yet deciphered. Taking this into account, and the fact that increased intestinal fatty acid absorption contributes to atherosclerosis onset and progression, this study aimed to evaluate the effect of TMA on fatty acid absorption in a cell line that mimics human enterocytes. Caco-2 cells were treated with TMA 250 μM for 24 h. Fatty acid absorption was assessed by measuring the apical-to-basolateral transport and the intracellular levels of BODIPY-C_12_, a fluorescently labelled fatty acid analogue. Gene expression of the main intestinal fatty acid transporters was evaluated by real-time quantitative reverse transcription PCR. Compared to control conditions, TMA increased, in a time-dependent manner and by 20–50 %, the apical-to-basolateral transport and intracellular levels of BODIPY-C_12_ fatty acid in Caco-2 cells. Fatty acid transport protein 4 (FATP4) and fatty acid translocase (FAT)/CD36 gene expression were not stimulated by TMA, suggesting that TMA-induced increase in fatty acid transport may be mediated by an increase in FAT/CD36 and/or FATP4 activity and/or fatty acid passive transport. This study demonstrated that TMA increases the intestinal absorption of fatty acids. Future studies are necessary to confirm if this may constitute a novel mechanism that partially explains the existing positive association between the consumption of a diet rich in TMA sources (e.g. red meat) and the increased risk of atherosclerotic diseases.

## Graphical abstract

**Figure:**
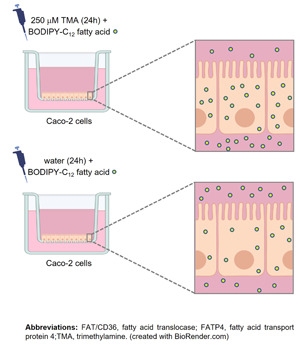

## Introduction

Due to its ability to metabolise non-digestible dietary compounds, the gut microbiota is capable of beneficially or detrimentally shaping host metabolic functions.^(1)^ A *bona fide* example illustrating gut microbiota's detrimental effects on host metabolism is the conversion of dietary L-carnitine and choline, mainly found in foods of animal origin (e.g. red meat), into the biogenic amine trimethylamine (TMA) by intestinal bacteria belonging to the genera *Acinetobacter*, *Serratia*, *Escherichia*, *Shigella*, *Klebsiella*, and *Providentia*.^(2)^ After its production, TMA is absorbed through the intestinal epithelium, further oxidised to trimethylamine *N*-oxide (TMAO) in the liver and then released into the systemic circulation.^(3)^ High plasma levels of TMAO have been positively associated with atherosclerosis progression and, consequently, with an increased risk of cardiovascular diseases.^(4,5)^ Although not fully elucidated, TMAO's proatherogenic effect seems to be explained by its capacity to inhibit the reverse transport of cholesterol from peripheral tissues to the liver, and to stimulate cholesterol uptake and accumulation within arterial macrophages leading to foam cells formation.^(4)^ Regarding TMAO's intestinal precursor, TMA, no previous studies, to our knowledge, have examined its potential contributory role to atherosclerosis via alterations in lipid metabolism.

Triacylglycerols (TGs), the main dietary lipids, are hydrolysed to fatty acids (FAs) and monoacylglycerols along the gastrointestinal tract, before being absorbed by enterocytes of the small intestinal epithelium.^(6)^ Within enterocytes, these hydrolysis products are recycled and used to resynthesize TGs, which are subsequently incorporated in apolipoprotein B48-containing lipoproteins, forming chylomicrons, and released into the circulation. Chylomicron components such as dietary FAs^(7)^ and apolipoprotein B48^(8,9)^ have been detected in human atherosclerotic plaques, and have been associated with their progression by promoting foam cells formation.^(10)^ For this reason, increased intestinal absorption of lipids, in particular FAs, has been considered an important risk factor for atherosclerosis.^(11)^ Taking this into account, and the fact that the role of the gut microbiota-derived metabolite TMA on intestinal lipid absorption has not been elucidated, this study aimed to evaluate the effect of TMA on fatty acid absorption in Caco-2 cells. Upon differentiation, these cells develop morphological and biochemical features similar to those of human small intestinal enterocytes, being thus suited to study lipid metabolism.^(12,13)^ Specifically, Caco-2 cells express genes encoding proteins involved in lipid absorption and transport,^(14)^ and chylomicrons production and secretion.^(15,16)^ Results obtained in this study will contribute to decipher the intestinal mechanisms by which TMA may potentially contribute to atherosclerosis.

## Materials and methods

### Caco-2 cell culture

The human epithelial cell line Caco-2 was obtained from the American-Type Culture Collection (ATCC37-HTB Rockville, MD, USA) and was used between passages number 27 and 56. Cell monolayers were cultured in minimum essential media at pH 7⋅4, supplemented with 15 % foetal bovine serum (FBS), 25 mM HEPES, 25 mM NaHCO_3_, 100 units/ml penicillin, 100 μg/ml streptomycin, and 0⋅25 μg/ml amphotericin B (all from Sigma-Aldrich, Madrid, Spain). Cells were maintained at 37 °C under a humidified atmosphere of 5 % CO_2_ – 95 % air. Culture media was changed every 2–3 d and cells were split every 7 d. For subculturing, cells were trypsinised using 0⋅25 % trypsin-ethylenediaminetetraacetic acid (EDTA) (Sigma-Aldrich), for 1 min at 37 °C, split 1:4, and seeded in plastic culture dishes (21 cm^2^; ∅ 52 mm; Orange Scientific, Braine-l'Alleud, Belgium). For the determination of cell viability, cells were seeded in 24-well plastic cell culture plates (1⋅93 cm^2^; ∅ 16⋅2 mm; Orange Scientific); for assessing fatty acid absorption and cell monolayer integrity, cells were seeded in 12-well Transwell inserts (polycarbonate membrane, 0⋅4 μm pore size, ∅ 12 mm; Corning Costar, NY, USA); and for RNA extraction, cells were seeded in 6-well plastic cell culture plates (9⋅6 cm^2^; ∅ 34⋅7 mm; Orange Scientific). After reaching 90–100 % confluence, cells were kept 11–12 d in culture before experiments, to allow spontaneous differentiation.

### TMA concentrations and incubation periods

Since no study appropriately determined TMA concentrations in the intestinal lumen of humans,^(2)^ the concentrations of TMA tested were chosen based on those found in faeces of healthy individuals, which ranged from 80 to 116 μM.^(17)^ Caco-2 cells were thus treated with physiologically relevant concentrations of TMA (Alfa Aesar, Kandel, Germany), i.e. 1, 10, 50, 100, or 250 μM for 2 or 24 h in FBS-free culture medium. The control of TMA was run in the presence of its solvent (water) at 0⋅01 % (v/v) concentration.

### Cell cytotoxicity assay

The effect of TMA on Caco-2 cells cytotoxicity was determined by the 3-(4,5-dimethyl thiazol-2-yl)-2,5-diphenyl tetrazolium bromide (MTT) assay.^(18)^ After treating cells with TMA, media was removed and each cell monolayer was incubated at 37 °C for 2 h with Hanks’ buffer (137 mM NaCl, 5 mM KCl, 0⋅8 mM MgSO_4_, 1⋅0 mM MgCl_2_, 0⋅33 mM Na_2_HPO_4_, 0⋅44 mM NaH_2_PO_4_, 0⋅25 mM CaCl_2_, 0⋅15 mM Tris-HCl, and 1⋅0 mM sodium butyrate (Sigma-Aldrich), pH = 7⋅4) containing 5 mg/ml MTT (Thermo Fisher Scientific, Leiden, The Netherlands). After this incubation period, MTT solution was carefully aspirated, cells were solubilised in 200 μl DMSO (Sigma-Aldrich) and diluted four times. The absorbance measured at 540 nm, which corresponds to purple formazan crystals produced due to mitochondrial dehydrogenases’ capacity to reduce MTT, was subtracted by the absorbance at 660 nm, which corresponds to unspecific MTT reduction. Both absorbances were measured using a SpectraMax i3x microplate reader (Molecular Devices, CA, USA). Results were expressed as % of control.

### Permeability assay

The effect of TMA on the permeability of Caco-2 cells monolayer was assessed by measuring the apical-to-basolateral passage of fluorescein sulphonic acid (FSA). Due to its ability to cross epithelia exclusively by the paracellular route (i.e. tight junctional spaces), FSA is considered a sensitive and valid biomarker of epithelial permeability^(19)^ and also integrity^(20)^ widely used in Caco-2 cells.^(21–24)^ Caco-2 cell monolayers were treated in the apical compartment of the Transwell insert with both TMA and 100 mg/ml FSA (Thermo Fisher Scientific) in the FBS-free culture medium. The FSA fluorescence (excitation wavelength at 485 nm and emission wavelength at 530 nm) was measured on the basolateral compartment using a SpectraMax i3x microplate reader (Molecular Devices). Results were expressed as % of control. Microscopic observation (Inverted light microscope ×400) of Caco-2 cells monolayer before measurement of FSA fluorescence showed no evidence of loss of monolayer intactness in control and TMA-treated cells.

### Fatty acid absorption assay

The effect of TMA on fatty acid absorption in Caco-2 cells was assessed by measuring the apical-to-basolateral transport and the intracellular levels of BODIPY-C_12_ fatty acid. The latter is a 12-carbon saturated fatty acid linked to the fluorophore BODIPY that, altogether, biologically resembles an 18-carbon long-chain fatty acid.^(25)^ Since BODIPY-C_12_ fatty acid has been demonstrated to undergo native-like transport and metabolism in human enterocytes,^(6,26)^ it is considered a well-established tool to study intestinal fatty acid absorption. After exposing cells to TMA in the apical compartment of Transwell inserts, media was removed, cells were washed twice with phosphate-buffered saline (PBS) (VWR, PA, USA), and incubated at 37 °C, in the apical compartment, with FBS-free culture media containing 10 μM BODIPY-C_12_ fatty acid (Thermo Fisher Scientific) and 0⋅1 % defatted bovine serum albumin (NZY Tech, Lisbon, Portugal). Fatty acid transport was assessed by measuring the appearance of BODIPY fluorescence in the basolateral compartment of Transwell inserts 0⋅5, 1, 2, 4, and 6 h after the apical incubation with BODIPY-C_12_ fatty acid, as described elsewhere.^(27)^ Intracellular fatty acid levels were assessed by measuring BODIPY fluorescence within cells 6 h after the incubation with BODIPY-C_12_ fatty acid. To do this, media from the apical compartment was removed, cells were carefully washed four to five times with PBS, and then lysed with 5 % IGEPAL (Sigma-Aldrich) in water. After that, cell lysates were centrifuged for 1 min at 8000 rpm and BODIPY fluorescence was measured in supernatants. BODIPY-C_12_ fluorescence (excitation wavelength at 578 nm and emission wavelength at 548 nm) was measured using a SpectraMax i3x microplate reader (Molecular Devices). Results were expressed in pmol of BODIPY-C_12_/mg of protein. The protein content of cell monolayers was determined with an EZQ protein quantification kit (Thermo Fisher Scientific), according to the manufacturer's instructions.

### RNA extraction and qRT-PCR

Total RNA was first extracted from TMA-treated Caco-2 cells using NZYol Isolation Reagent and then treated with DNase I (NZY Tech, Lisbon, Portugal), following the manufacturer's instructions. For cDNA synthesis, 1 μg of total RNA was reverse transcribed using the NZY First-Strand cDNA Synthesis Kit, following the manufacturer's instructions. Each individual real-time quantitative reverse transcription polymerase chain reaction (qRT-PCR) was performed using: 0⋅2 μM of each primer and FastStart SYBR Green Master (Roche, IN, USA) according to manufacturer instructions. Thermocycling conditions were the following: heat activation (50 °C for 2 min), incubation (95 °C for 10 min), denaturation, annealing, and elongation (95 °C for 15 s and 60 °C for 1 min) repeated 45 times and a melting curve programme (95 °C for 15 s, 60 °C for 15 s, and 95 °C with a heating rate of 0⋅1 °C/s and continuous fluorescence measurement). Primer pairs used for amplification, their sequences and annealing temperatures are listed in Table 1. PCR was carried out in duplicate for each gene using a LightCycler® 96 device (Roche, NJ, USA). Data were analysed using the LightCycler® 96 analysis software, and gene expression was normalised for the housekeeping gene hypoxanthine guanine phosphoribosyltransferase (HPRT). HPRT demonstrated to be an appropriate housekeeping gene since its mean cycle threshold values in control and TMA-treated cells were comparable (26⋅16 ± 0⋅18 cycles *v.* 26⋅10 ± 0⋅10 cycles) and were of similar order of magnitude of that of CD36 and FATP4 in all samples (26⋅13 ± 0⋅1 cycles *v.* 28⋅04 ± 0⋅09 cycles *v.* 25⋅37 ± 0⋅15). Moreover, HPRT is considered a reference gene widely used to normalise fatty acid transporters gene expression data in several types of mammalian cell lines (including Caco-2).^(28)^

**Table 1.** Primer sequences and annealing temperatures used for qRT-PCR

Gene name	Primer sequence (5′ to 3′)	Annealing temperature (°C)
FAT/CD36	GGTACAGATGCAGCCTCATTTC (F) GCAAAGGCCTTGGATGGAAG (R)	60
FATP4	CTACCTTACTGGTGATGTGCTG (F) GCGGCTGAGTGTGCCTTCCAC (R)	60
HPRT	TTTATGTCCCCTGTTGACTGG (F) TGCTGACCTGCTGGATTACA (R)	59

FAT/CD36, fatty acid translocase; FATP4, fatty acid transport protein 4; HPRT, hypoxanthine guanine phosphoribosyltransferase; (F), forward; (R), reverse.

### Statistical analysis

Statistical analysis was performed using GraphPad Prism V8.0 (GraphPad Software, Inc., USA). Data are shown as mean ± standard error of the mean (sem). *n* represents the number of replicates of at least two different days of experiments. The statistical significance of the difference between the two groups was evaluated by the Student's *t* test and the statistical difference between three or more groups was evaluated by one-way ANOVA with Tukey *post-hoc* testing. Differences were considered to be significant when *P* < 0⋅05.

## Results

### TMA is not cytotoxic to Caco-2 cells

**Fig. 1.**
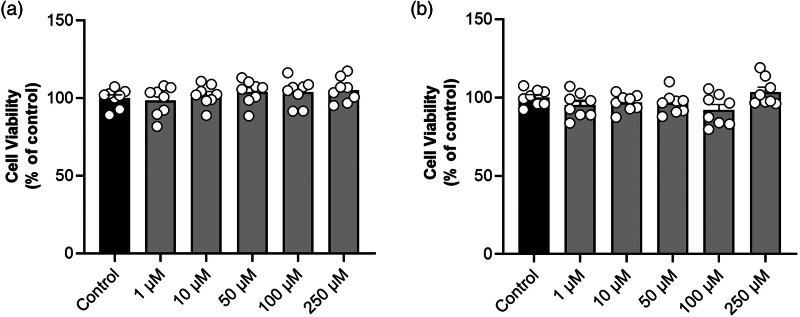
Trimethylamine is not cytotoxic to Caco-2 cells. Effect of (a) a 2- and (b) a 24-h exposure to different concentrations of trimethylamine on Caco-2 cells cytotoxicity, determined by the MTT assay. Data are shown as mean ± sem (*n* 8).

To assess the cytotoxic potential of TMA on Caco-2 cells, the MTT assay was performed. Concentrations of TMA of the same order of magnitude as those found in human faeces (see Materials and methods) were not cytotoxic to Caco-2 cells after a 2- or 24-h treatment (Fig. 1). Based on this, Caco-2 cells were treated with the highest concentration (250 μM) and exposure time (24 h) of TMA in the following experiments.

### TMA maintains Caco-2 cells monolayer integrity

**Fig. 2.**
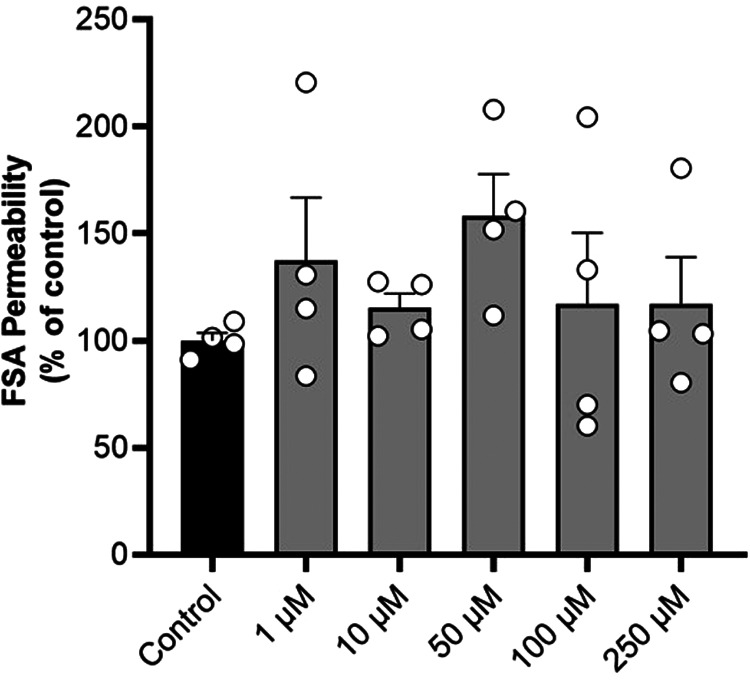
Trimethylamine maintains Caco-2 cells monolayer integrity. Effect of a 24-h exposure to trimethylamine 250 μM on Caco-2 cells monolayer integrity, determined by measuring the apical-to-basolateral passage of fluorescein sulfonic acid (FSA). Data are shown as mean ± sem (*n* 4).

To assess whether TMA alters Caco-2 cells monolayer integrity, the apical-to-basolateral passage of FSA was measured following TMA treatment. When compared to control conditions, TMA did not alter FSA passage suggesting that it maintains cell monolayer integrity (Fig. 2).

### TMA increases fatty acid absorption in Caco-2 cells

**Fig. 3.**
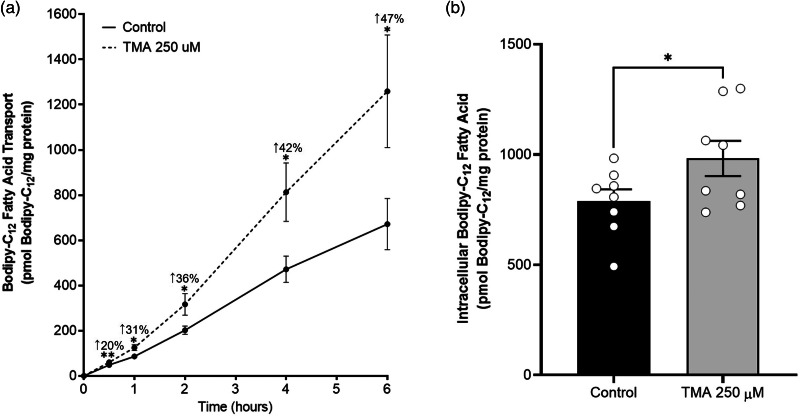
Trimethylamine increases fatty acid absorption in Caco-2 cells. Effect of a 24-h exposure to trimethylamine 250 μM on the transport (a) and intracellular levels (b) of fatty acids in Caco-2 cells. Fatty acid transport was determined by measuring the apical-to-basolateral passage of BODIPY-C_12_ fatty acid. Intracellular fatty acid levels were determined by measuring BODIPY-C_12_ fatty acid within cells. In panel (a), the increase (in % of control) of BODIPY-C_12_ fatty acid transport for each time point in trimethylamine-treated cells is shown. Data are presented as mean ± sem (*n* 8). *P < 0⋅05 and **P < 0⋅01 *v.* controls.

After Caco-2 cells exposure to TMA, fatty acid absorption was assessed by measuring the apical-to-basolateral transport and intracellular levels of BODIPY-C_12_ fatty acid, a fluorescently labelled long-chain fatty acid analogue. This fatty acid has been previously shown to be absorbed, incorporated in TGs and apolipoprotein B48-containing lipoproteins, and subsequently secreted as part of chylomicrons by Caco-2 cells.^(29)^ Our results showed that the transport of BODIPY-C_12_ fatty acid steadily increased in a time-dependent and non-saturable manner in both control and TMA-treated cells 0⋅5, 1, 2, 4, and 6 h after its addition to the apical compartment (Fig. 3(a)). However, at all-time points, the magnitude of this increase was 20–50 % higher in TMA-treated cells (Fig. 3(a)). Regarding intracellular BODIPY-C_12_ fatty acid, their levels were assessed at the end of transport experiments, i.e. 6 h after BODIPY-C_12_ fatty acid addition to the apical compartment of Caco-2 cells. Intracellular BODIPY-C_12_ fatty acid levels were 20 % higher in cells exposed to TMA compared to those exposed to control conditions (981⋅9 ± 79⋅80 pmol of BODIPY-C_12_/mg protein *v.* 788⋅3 ± 53⋅92 pmol of BODIPY-C_12_/mg protein) (Fig. 3(b)).

### TMA alters fatty acid transporters gene expression

**Fig. 4.**
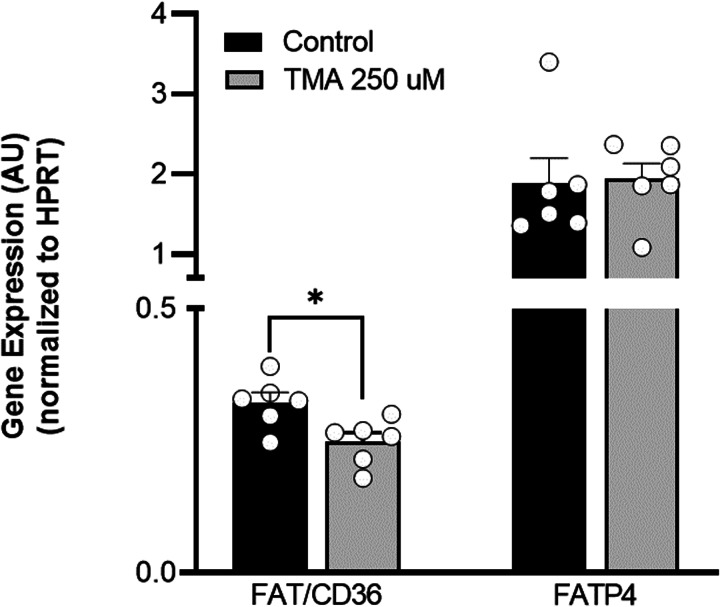
Trimethylamine alters fatty acid transporters gene expression. Effect of a 24-h exposure to trimethylamine 250 μM on fatty acid translocase (FAT)/CD36 and fatty acid transport protein 4 (FATP4) gene expression in Caco-2 cells. Relative expression of these genes was evaluated by quantitative qRT-PCR. Results obtained were normalized for the housekeeping gene hypoxanthine guanine phosphoribosyltransferase (HPRT). Data are expressed as mean ± sem (*n* 6). *P < 0⋅05 *v.* controls. AU, arbitrary unit.

To investigate the mechanisms underlying the TMA-induced increase in fatty acid absorption, gene expression of the main intestinal fatty acid transporters, fatty acid transport protein 4 (FATP4),^(6)^ and fatty acid translocase (FAT)/CD36^(30)^ were analysed in Caco-2 cells by qRT-PCR. Although TMA was found to decrease FAT/CD36 expression by 23 % compared to control conditions, it did not change FATP4 expression (Fig. 4).

## Discussion

Increasing evidence implicates gut microbiota-derived metabolites in the development of cardiovascular diseases (reviewed by^(1,31)^). Of those, particular attention has been dedicated to TMAO, since its blood levels have been reported to be an independent predictor of atherosclerosis development in humans.^(4,5,32)^ TMAO is produced in the liver from its intestinal precursor, TMA, which is generated from dietary L-carnitine and choline, mostly present in red meat, by gut bacteria metabolism.^(2)^ In contrast to TMAO, the role of TMA in atherosclerosis has not been yet deciphered. Taking this into account, and the fact that increased intestinal absorption of dietary lipids,^(8,11)^ in particular FAs,^(7)^ contribute to atherosclerosis onset and progression, this study aimed to assess the effect of TMA on intestinal fatty acid absorption. Using a cell model that mimics human small intestinal enterocytes,^(12,13)^ this study showed that TMA increased fatty acid absorption by increasing the apical-to-basolateral transport and intracellular accumulation of BODIPY-C_12_ fatty acid in Caco-2 cells. Although further animal and clinical confirmatory studies are needed, these results raise the hypothesis that TMA may be regarded as a putative proatherogenic risk factor. This hypothesis has been partially supported by Wang *et al.,*^(33)^ as they showed that inhibition of TMA production by the gut microbiota was able to prevent atherosclerosis development in atherosclerosis-prone *Apoe*^−/−^ mice. Despite these observations, authors did not distinguish the impact on atherosclerosis resulting from the inhibition of TMA production in the absence and presence of circulating levels of TMAO.^(33)^

FAT/CD36^(34)^ and FATP4^(6)^ are highly expressed in the apical membrane of enterocytes, being the main transporters involved in dietary fatty acid absorption. In this study, TMA did not change FATP4 and even decreased FAT/CD36 gene expression in Caco-2 cells. The discrepancy observed between these results and those of BODIPY-C_12_ fatty acid absorption, suggests that TMA may increase transport and intracellular levels of FAs by inducing (i) the activity (i.e. transport capacity), rather than gene expression, of FAT/CD36 and/or FATP4 and/or (ii) the protein-independent, non-saturable and passive transport of FAs. However, at the same time, Caco-2 cells may also develop a transcriptional compensatory mechanism, particularly at the level of FAT/CD36 gene, which may reflect the capacity of the methyl group donor TMA^(35)^ to induce DNA methylation of FAT/CD36 gene, thereby decreasing its transcription and, consequently, its mRNA expression levels.^(36)^ Considering that gene expression of other less abundant intestinal fatty acid transporters was not assessed, it cannot be excluded that TMA may alter their expression, namely of plasma membrane fatty acid-binding protein (FABPpm), FATP1-3, 5, or 6, or fatty acid-binding protein (FABP). Although detected in primary enterocytes, these transporters have a significantly lower physiological contribution to the absorption of dietary long-chain FAs compared with that of FATP4 and CD36.^(6,34)^ Moreover, these two transporters were shown to be highly expressed in Caco-2 cells,^(14,28,37)^ corroborating their significant contribution to long-chain FAs absorption also in this cell line. On the other hand, a TMA-induced increase in fatty acid transport through paracellular diffusion was excluded, since the apical-to-basal passage of FSA, a marker of paracellular transport,^(19)^ was similar in both control and TMA-treated Caco-2 cells.

To the best of our knowledge, this is the first study to assess the effect of TMA on intestinal lipid absorption. A previous study analysed the effect of a diet supplemented with TMAO on intestinal absorption of cholesterol in *Apoe*^−/−^ mice. In comparison with a normal diet, the TMAO-supplemented diet reduced the intestinal absorption of cholesterol. However, these results were not the explanation for the proatherogenic effect of the TMAO-supplemented diet observed in *Apoe*^−/−^ mice, but rather its capacity to stimulate cholesterol uptake in arterial macrophages by upregulating gene expression of cholesterol transporters, i.e. CD36 and scavenger receptor A, at their surface.^(4)^ These results, together with the ones from this study, suggest that TMAO and TMA regulate the transport of atherogenic lipids in a tissue-specific manner.

In conclusion, this study demonstrated that TMA increases intestinal fatty acid absorption by increasing the transepithelial transport and intracellular levels of FAs in human enterocytes. Although this is a small-scale *in vitro* study, it raises important implications that deserve to be confirmed in future studies. Namely, if the TMA-induced increase in intestinal fatty acid absorption may constitute a novel mechanism that partially explains the positive association observed between the consumption of a diet rich in L-carnitine and choline sources, in particular red meat^(38)^ and supplements,^(4)^ and an increased risk of atherosclerotic cardiovascular diseases.
